# Early experience with sotatercept in adult patients with repaired and unrepaired congenital heart disease and pulmonary hypertension

**DOI:** 10.1016/j.ijcchd.2026.100721

**Published:** 2026-10-14

**Authors:** Hans Gao, Richard Channick, Rajan Saggar, Michael Troy, Jeannette Lin, Gentian Lluri, Leigh Reardon, Jamil Aboulhosn

**Affiliations:** a Ahmanson/UCLA Adult Congenital Heart Disease Center, Division of Cardiology, Department of Medicine, David Geffen School of Medicine at UCLA, Los Angeles, CA, USA; b Division of Pulmonary and Critical Care Medicine, Department of Medicine, David Geffen School of Medicine at UCLA, Los Angeles, CA, USA

**Keywords:** Sotatercept, Pulmonary arterial hypertension, Adult congenital heart disease, Activin signaling inhibitor, Right ventricular remodeling, Eisenmenger syndrome

## Abstract

**Background:**

Pulmonary arterial hypertension associated with congenital heart disease (ACHD-PAH) is a condition underrepresented in initial clinical trials investigating sotatercept, a first-in-class activin signaling inhibitor. Patients with unrepaired defects or complex congenital heart disease were excluded from these trials. Here, we describe real-world outcomes in a cohort of ACHD-PAH patients treated with sotatercept.

**Methods:**

A single-center retrospective review was performed of 14 adults with ACHD-PAH initiated on sotatercept between May 2024 and January 2026. Patients had heterogeneous congenital anatomy; with half having unrepaired defects. Paired pre- and post-treatment measures were compared, and each patient served as their own control, over a median follow-up of 542 days.

**Results:**

Eleven of 14 patients (79%) continued therapy. Three patients discontinued: one for epistaxis and two for clinical deterioration leading to heart-lung transplant listing. Adverse effects occurred in 50% of patients, most commonly epistaxis (43%). Hemoglobin increased by a median of 1.5 g/dL (p = 0.002). Symptomatic improvement was reported by 79%, and 29% improved by at least one WHO functional class. Exertional oxygen-saturation nadir improved in 71%. Echocardiography showed favorable subpulmonary ventricular remodeling, with increased fractional area change (+5.0%, p = 0.020) and reductions in right ventricular basal, mid-chamber, and longitudinal dimensions (all p ≤ 0.03). Lack of response was associated with advanced disease, pulmonary veno-occlusive physiology, and systemic ventricular dysfunction.

**Conclusions:**

In this cohort of ACHD-PAH patients, sotatercept was generally well-tolerated and was associated with symptomatic improvement and echocardiographic signs of favorable ventricular remodeling.

## Graphical abstract

**Figure:**
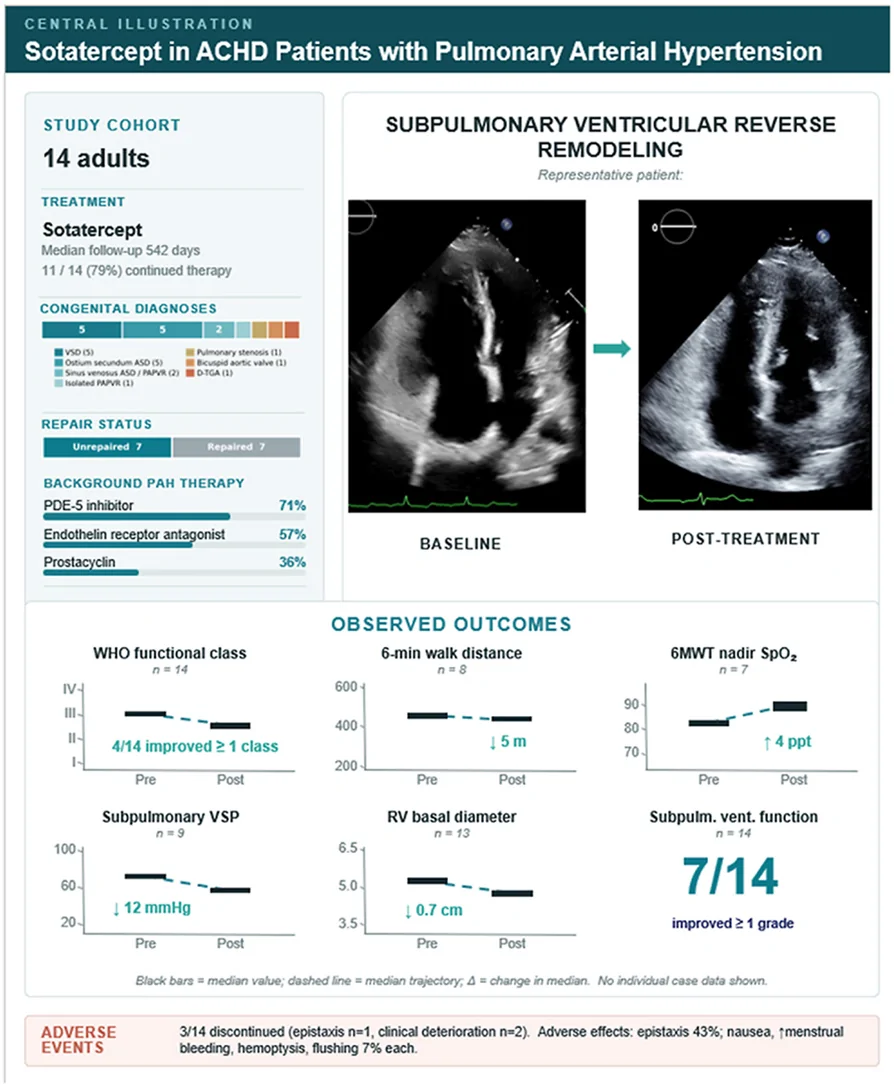

## 1 Background

Pulmonary arterial hypertension associated with congenital heart disease (ACHD-PAH) is a challenging and heterogenous subset of Group 1 PAH, often associated with abnormal systemic ventricular function which may produce a mixed picture of Group 1 and Group 2 PAH. Survival in ACHD-PAH is better than in idiopathic PAH (5-year survival ∼76% vs. 54%) but remains markedly worse than in ACHD patients without PAH [1].

Sotatercept, a first-in-class activin modulator, has shown substantial improvements in pulmonary vascular resistance (PVR), exercise capacity, and clinical outcomes in randomized clinical trials [2]. However, ACHD-PAH patients were underrepresented in these trials, comprising only 5% (n = 16) of the STELLAR trial, and 5.2% (n = 9 patients) in the Zenith trial [2], [3]. Here, we describe our real world experience of fourteen adults with ACHD-PAH initiated on sotatercept for progressive symptomatic decline.

## 2 Methods

### 2.1 Patient selection

We performed a retrospective chart review of all adults with congenital heart disease and pulmonary arterial hypertension (ACHD-PAH) who were initiated on sotatercept therapy between May 2024 and January 2026 at the Ahmanson/UCLA Adult Congenital Heart Disease Center. Institutional review board approval was obtained prior to data collection. In thirteen out of fourteen cases, sotatercept was prescribed for symptomatic, persistent pulmonary hypertension despite optimized medical therapy. In one case, sotatercept was prescribed at time of initial diagnosis as part of a multi-drug regimen. Clinical notes, laboratory data, and imaging studies obtained before and after sotatercept initiation were systematically reviewed. All surviving patients included in the analysis continue to receive longitudinal care at the Ahmanson/UCLA Adult Congenital Heart Disease Center. Immediate and short-term clinical outcomes were reviewed for analysis.

### 2.2 Follow-up evaluation

Adverse effects were attributed to sotatercept when the treating clinician and study reviewer were in agreement that the event was likely related to therapy. Hemoglobin was monitored at baseline, at one to two weeks after initiation of medication, and at subsequent clinic visits. Patients were additionally evaluated for interval changes in symptoms and World Health Organization (WHO) functional class status.

### 2.3 Statistical analysis

Data was collected retrospectively. Continuous variables are reported as median (interquartile range [IQR]) and categorical variables as counts (percentages). Paired pre- and post-treatment values were compared using the exact Wilcoxon signed-rank test, with each patient serving as their own control; a two-sided p < 0.05 was considered statistically significant. Given the small sample size, p-values are meant to be descriptive and were not adjusted for multiple comparisons. Right ventricular echocardiographic measures were analyzed only amongst patients whose subpulmonary ventricle was a morphologic right ventricle (n = 13). Analyses were performed in Python.

## 3 Results

### 3.1 Baseline characteristics

The study population consisted of 14 patients. Baseline characteristics are summarized in Table 1. The median age was 41 years (range 23 to 61), and 10 (71%) patients were female. Median weight was 60.9 kg (IQR 49.5-70.1) with a median BMI of 22.6 kg/m^2^ (20.9-25.0).

**Table 1:** Baseline characteristics.

**Baseline Characteristics**	
Female, n (%)	10 (71%)
Age (years)	41 (23–61)
Weight (kg)	60.9 (49.5–70.1)
BMI (kg/m^2^)	22.6 (20.9–25.0)
**Congenital heart diagnoses**^a^	
VSD	5 (36%)
Ostium secundum ASD	5 (36%)
Sinus Venosus ASD with PAPVR	2 (14%)
Isolated PAPVR	1 (7%)
Pulmonary Stenosis	1 (7%)
Bicuspid Aortic Valve	1 (7%)
D-Transposition of the Great Arteries	1 (7%)
**Repair Status**	
Unrepaired	7 (50%)
Repaired	7 (50%)
**Systemic ventricle systolic function**	
Normal	13 (93%)
Mild	0
Moderate	0
Severe	1 (7%)
**Subpulmonic ventricle systolic function**	
Normal	3 (21%)
Mild	3 (21%)
Moderate	4 (29%)
Severe	4 (29%)
**WHO Functional Class**	
I	0
II	3 (21%)
III	10 (71%)
IV	1 (7%)
**Background Therapy**	
Tadalafil	10 (71%)
Macitentan	8 (57%)
Inhaled Treprostinil	5 (36%)
Ambrisentan	3 (21%)
Sildenafil	2 (14%)
Selexipag	2 (14%)
SubQ Treprostinil	2 (14%)
IV Treprostinil	2 (14%)
Bosentan	1 (7%)
Riociguat	1 (7%)
Oral Treprostinil	1 (7%)

a Patients may have more than one congenital heart diagnosis. Age is reported as median (range). Weight and BMI reported as median (IQR). Categorical variables are (n%).

Patients had a wide spectrum of congenital heart diagnoses, most commonly ventricular septal defect (36%) and ostium secundum defect (36%), followed by sinus venosus atrial septal defect with partial anomalous pulmonary venous return (14%). Additional diagnoses included pulmonary stenosis, bicuspid aortic valve, and D-transposition of the great arteries.

Half of the cohort (50%) were unrepaired. Systemic ventricular systolic function was preserved in nearly all patients (93%), however subpulmonary ventricular dysfunction was common, with 57% demonstrating moderate or severe dysfunction by echocardiography.

The majority of patients were markedly symptomatic at baseline: 71% were WHO functional class III and 7% were class IV. One patient was initiated on sotatercept after a new diagnosis of pulmonary hypertension; the other 13 patients were already being treated with pulmonary vasodilators, with the three most common therapies being tadalafil (71%), macitentan (57%) and inhaled treprostinil (36%).

## 4 Effects of treatment

Over a median follow-up of 542 days (IQR 380-674), 11 of 14 patients (79%) tolerated sotatercept therapy without discontinuation (Table 2). Three patients (21%) discontinued treatment, one due to epistaxis despite nasal cautery, and the other two due to clinical deterioration despite sotatercept initiation followed by urgent admission for heart-lung transplant listing. Adverse effects were reported in 50% of patients, most commonly epistaxis (43%). Less frequent events included nausea (7%), increased menstrual bleeding (7%), hemoptysis (7%), and flushing (7%). Hemoglobin levels demonstrated a median paired increase of 1.5 g/dL (IQR 0.5 to 2.3, p = 0.002).

**Table 2:** Clinical and echocardiographic outcomes before and after sotatercept therapy (n = 14).

Variable	n	Baseline, median (IQR)^a^	Within-patient change, median (IQR)^a^	p-value^b^
**Functional and laboratory outcomes**				
Hemoglobin (g/dL)	14	14.1 (12.5–15.1)	+ 1.5 (0.5 to 2.3)	**0.002**
**WHO functional class**	**14**	≥1 class better: 4/14 (29%)		0.125
Class II		3 (21%)	7 (50%)	
Class III		10 (71%)	7 (50%)	
Class IV		1 (7%)	0 (0%)	
6-min walk distance (m)	8	450 (386–533)	−5 (−16 to 24)	1.000
6MWT resting SpO_2_ (%)	7	96 (93–97)	+2 (−1 to 5)	0.375
6MWT nadir SpO_2_ (%)	7	82 (75–96)	+4 (−2 to 14)	0.297
Self-reported symptom improvement	14	11/14 (79%)		
***Echocardiographic outcomes***				
Subpulmonary ventricular systolic pressure (mmHg)	9	71 (58–83)	−12 (−21 to −3)	0.074
RV fractional area change (%)	13	25 (17-37)	+5 (0 to 11)	**0.020**
TAPSE (cm)	13	1.4 (1.2-2.0)	+0.4 (−0.3 to 0.5)	0.110
Tissue-Doppler S’ (cm/s)	12	9.6 (8.2-12.6)	+0.2 (−1.3 to 1.3)	0.85
RV basal diameter (cm)	13	5.2 (4.6–5.9)	−0.7 (−1.0 to 0.0)	**0.021**
RV mid-chamber diameter (cm)	13	4.8 (4.1–5.1)	−0.9 (−1.4 to −0.1)	**0.027**
RV longitudinal length (cm)	13	9.9 (8.7–10.1)	−1.0 (−1.9 to 0.1)	**0.021**
**Subpulmonary ventricular size**	**14**	≥1 grade better: 8/14 (57%)		**0.020**
Normal/upper normal		0 (0%)	6 (43%)	
Mildly enlarged		3 (21%)	3 (21%)	
Moderately enlarged		5 (36%)	2 (14%)	
Severely enlarged		6 (43%)	3 (21%)	
**Subpulmonary ventricular function**	**14**	≥1 grade better: 7/14 (50%)		**0.008**
Normal/low normal		3 (21%)	8 (57%)	
Mildly reduced		3 (21%)	1 (7%)	
Mild-to-moderately depressed		0 (0%)	1 (7%)	
Moderately reduced		4 (29%)	4 (29%)	
Severely reduced		4 (29%)	0 (0%)	

Values for continuous variables are median (IQR). Categorical variables are n (% of denominator).

Echocardiographic RV size and function reflect qualitative reader interpretation; upper normal and low normal were treated as within-normal borderline categories. Subpulmonary ventricular systolic pressure was excluded when the subpulmonary atrioventricular valve regurgitation jet was not adequately seen.

**Abbreviations:** IQR, interquartile range; RV, right ventricle; SpO_2_, peripheral oxygen saturation; WHO-FC, World Health Organization functional class; 6MWT, 6-min walk test.

a For categorical variables (WHO functional class, subpulmonary ventricular size and function), the Baseline and Within-patient change columns show the number (%) of patients in each category before and after sotatercept, respectively. The paired result for these variables is the proportion that improved by ≥ 1 grade/class, with exact Wilcoxon p-value shown on the variable header row.

b Wilcoxon signed-rank test (exact method) on paired observations; bolded when p < 0.05. The analysis is descriptive and exploratory; no correction for multiple comparisons was applied. Pairs with a missing pre- or post-sotatercept measurement were excluded per variable.

The majority of patients experienced symptomatic improvement following sotatercept initiation, with 79% self-reporting improvement in symptoms and 29% with an improvement in WHO functional class. Six minute walk test distance was available in 8 patients and showed no consistent change (median -5m), increasing in 4 and decreasing in 4 patients. Six minute walk test oxygen saturation nadir improved in 71% of patients (5 of 7). Echocardiographic assessment demonstrated structural and functional changes in the subpulmonary ventricle**,** which was the morphologic right ventricle in 13 patients and the morphologic left ventricle in one patient with D-transposition of the great arteries following atrial switch repair. Qualitatively, subpulmonary ventricular function improved in 50% of patients, and subpulmonary ventricular size improved in 57%. For those with subpulmonary right ventricles, quantitative measurements showed modest changes in function, including a median increase in fractional area change of 5.0% (IQR 0.0 to 11, p = 0.020), TAPSE by 0.4 cm (IQR -0.3 to 0.5, p = 0.110), and tissue-Doppler S’ by +0.2 cm/s (IQR -1.3 to 1.3, p = 0.85). Reductions in right ventricular dimensions were observed across multiple forms of measurements, including basal diameter (−0.7 cm, IQR -1.0 to 0.0, p = 0.021), mid-chamber diameter (−0.9 cm, IQR -1.4 to −0.1, p = 0.027), and longitudinal length (−1.0 cm, IQR -1.9 to 0.1, p = 0.021). Estimated subpulmonary ventricular systolic pressure decreased by a median of 12 mmHg (IQR –21 to −3 mmHg, p = 0.074) (Fig. 1).

**Fig. 1:**
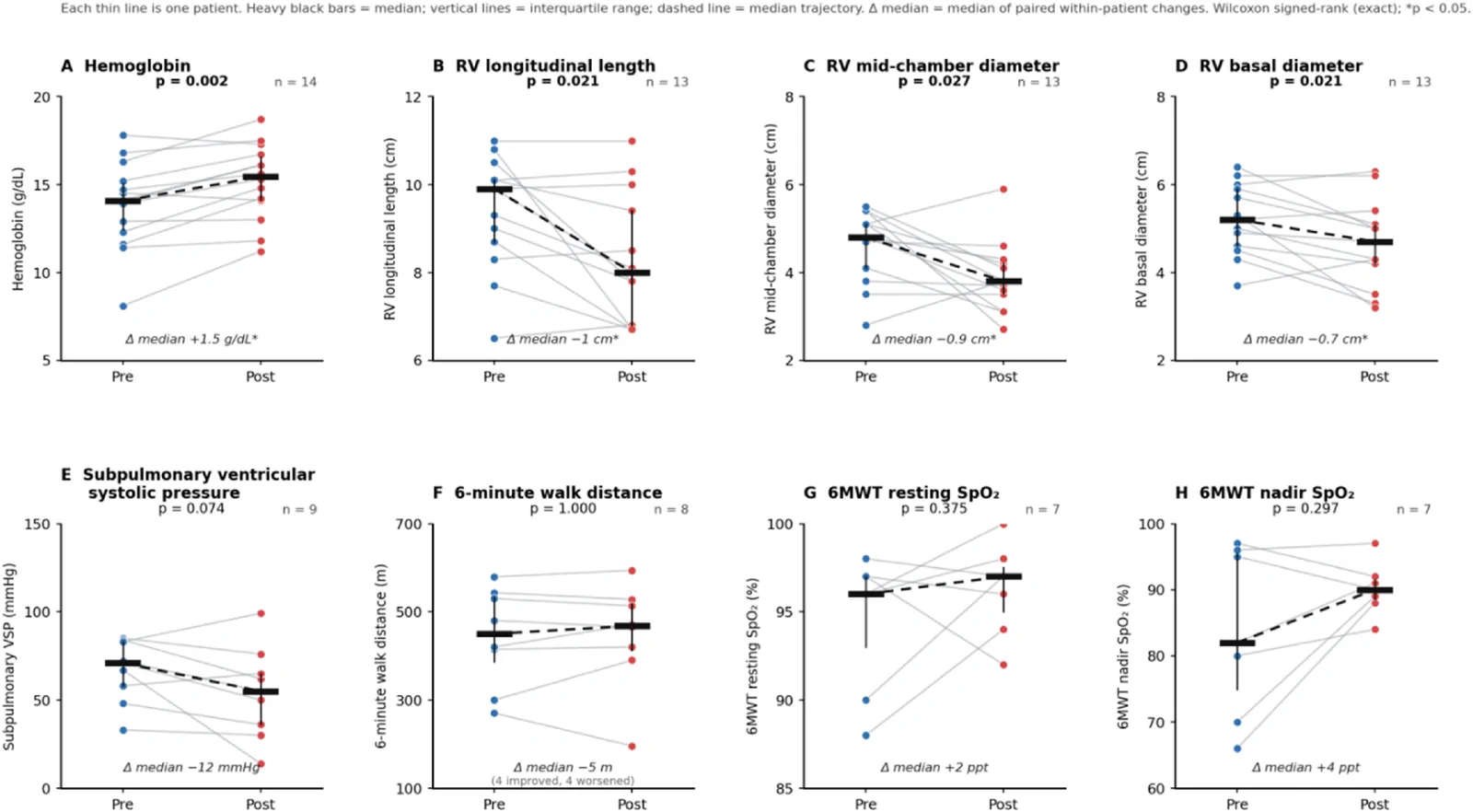
Paired clinical and echocardiographic outcomes before and after sotatercept.

## 5 Illustrative cases: non-responders

Therapy discontinuation occurred in three patients (Cases 4, 11, 13) and are described in more detail below. Further details can be viewed in Supplemental Table 1.

Case 4 is a 39 year old woman with an unrepaired ostium secundum ASD on background therapy of subcutaneous treprostinil, sildenafil and bosentan. She had a chronic history of epistaxis requiring nasal cautery prior to sotatercept initiation. Sotatercept was discontinued 20 weeks after initiation due to recurrence of her epistaxis despite repeat nasal cautery. Her epistaxis improved after discontinuation.

Case 11 is a 26 year old man with a repaired VSD, bicuspid aortic valve (BAV), and bioprosthetic aortic valve replacement, who presented with acute decompensated heart failure. Baseline TTE showed moderate-to-severe aortic stenosis (peak gradient 44 mmHg, mean gradient 24 mmHg, aortic valve area 0.7 cm^2^) and flail aortic leaflet resulting in severe regurgitation. High-risk transcatheter aortic valve replacement (TAVR) was considered, however the decision was made to first optimize him on PAH therapy prior to intervention. He was discharged on macitentan, tadalafil, and inhaled treprostinil, with the addition of sotatercept as an outpatient. After 10 months of sotatercept therapy, he did not have clinical improvement and was admitted after an episode of hemoptysis. Sotatercept was discontinued while he was inpatient, and he subsequently underwent combined heart and lung transplant. He is currently doing well in the outpatient setting. Explant pathology of his native lungs showed pulmonary capillary hemangiomatosis consistent with pulmonary veno-occlusive disease; a phenotype that does not typically respond favorably to pulmonary vasodilator therapy.

Case 13 is a 61 year old woman with D-transposition of the great arteries status post atrial switch and severe pulmonary hypertension, on background therapy of inhaled treprostinil and tadalafil. TTE showed severely reduced subpulmonary LV function with EF of 20-25%, as well as severely reduced systemic RV function. She was evaluated for combined heart and lung transplantation – however after extensive discussion, she had a strong preference to first trial sotatercept for several months as an outpatient, and to defer transplant evaluation. After two months she did not show signs of clinical improvement and was admitted due to decompensated heart failure and completion of her transplant evaluation. She developed cardiogenic shock with multiorgan failure and was declined for transplant listing due to severity of critical illness, and died during that hospitalization. Her family declined autopsy.

## 6 Illustrative cases: clinical responders

Case 3 is a 38 year old woman with newly diagnosed ostium secundum ASD who transferred from an outside hospital with pulmonary hypertension crisis and acute RV failure, with initiation of VA ECMO shortly after arrival. She had findings of right-to-left shunting on echocardiography, extensive in situ PA thrombosis on CT angiography, and clubbed digits. Pulmonary hypertension therapy was initiated while she was on VA ECMO, which included tadalafil and IV epoprostenol that was subsequently transitioned to subcutaneous treprostinil. She was successfully decannulated from VA ECMO and several days later started on sotatercept as an inpatient. At 3 month outpatient follow up she had marked improvement in function, with ability to walk half an hour without dyspnea and return to work.

Case 5 is a 33 year old woman with a large, non-restrictive VSD and reversed shunt physiology with WHO III symptoms, maintained on macitentan, tadalafil and inhaled treprostinil. At six month follow up post-sotatercept, she reported an improvement in symptoms of daytime fatigue and dyspnea on exertion. She reported mild worsening of her chronic epistaxis after initiation as her only noted adverse effect. Hemoglobin increased modestly from 16.8 g/dL to 17.5 g/dL. TTE demonstrated unchanged normal RV function and mildly increased size by basal diameter (3.7 cm to 4.3 cm) and longitudinal length (6.5 cm to 6.8 cm).

Case 10 is a 44 year old woman with congenital pulmonary valve stenosis status post transcatheter pulmonary valve replacement with systemic PA pressures of 95/37 mmHg by right heart catheterization had severe dyspnea despite triple therapy including tadalafil, macitentan, and subcutaneous treprostinil. Lung transplantation was being considered. Following sotatercept initiation, she had dramatic symptomatic improvement and was able to walk up to 7 miles daily. Echocardiography showed improved RV function (TAPSE 1.6 cm, FAC 32%, improved from 1.1 cm to 18% respectively).

## 7 Discussion

This study describes the outcomes of a cohort of diverse adult congenital heart disease patients with severe pulmonary hypertension who were treated with sotatercept (**Central Illustration**). The available endpoints were primarily echocardiographic and patient-reported, as most patients had not yet undergone follow up cardiac catheterization. Eleven out of fourteen patients reported subjective symptomatic improvement, and several had corresponding improvements in echocardiographic markers of subpulmonary systolic function and estimated systolic pressure.

Of the three patients who discontinued therapy, only one was due to adverse reaction (exacerbation of chronic epistaxis). The other two (Case 11 and 13) were discontinued due to lack of efficacy. Notably, case 13 was the only patient in the cohort with systemic ventricular dysfunction, raising the possibility that this subgroup may be less responsive to sotatercept. Case 11 was found on explant to have pulmonary veno-occlusive disease, a phenotype known to respond poorly to pulmonary vasodilators. Case 13 was trialed on sotatercept for only two months before being admitted for clinical decline, which may have been insufficient treatment duration to detect therapeutic effect. Both cases also were admitted inpatient for transplant listing, suggesting their disease may have been more advanced than the other patients in the cohort. While these observations are limited to a small number of non-responders, systemic ventricular dysfunction, veno-occlusive physiology, and advanced disease, may warrant consideration when selecting ACHD-PAH patients for sotatercept and should be investigated further.

For the other eleven patients, sotatercept therapy was overall well-tolerated, with epistaxis being the most common side effect in 43% of patients. Both patients with reversed shunt physiology were able to tolerate sotatercept, and one of the patients was initiated shortly after VA ECMO decannulation for cardiogenic shock in the setting of pulmonary hypertension crisis. It bears noting that the erythropoietic effect of sotatercept should be closely attended in the ACHD-PAH population. Several patients in this cohort had baseline erythrocytosis from chronic cyanosis (a population underrepresented or excluded from previous sotatercept trials) [4], however none experienced hyperviscosity symptoms such as headache or visual changes. Careful monitoring of hemoglobin levels and hyperviscosity symptoms remains important, particularly in patients with chronic cyanosis.

Notably, both the STELLAR and ZENITH trials limited enrollment of CHD-associated PAH to patients with systemic-to-pulmonary shunts that had already been repaired. Patients with unrepaired shunts, including those with reversed-shunt physiology, were excluded. In our cohort, half (7/14) had unrepaired defects, including two with reversed-shunt physiology, both of whom tolerated sotatercept. Importantly, these patients were comanaged at a specialized ACHD center with an experienced PAH center. Integration of sotatercept into ACHD-PAH care should be done thoughtfully, with attention paid to any potential interaction with residual shunts, valve dysfunction, and unique anatomies. Identifying patient-specific characteristics predictive of responsiveness (or non-responsiveness) to sotatercept remains important for future investigation.

This series has several limitations. It is a small, retrospective study conducted at a single referral center. Follow-up imaging timing was not standardized. As previously noted, most patients had not yet undergone follow up cardiac catheterization to assess hemodynamic response. However, the trend of clinical improvement and echo-derived surrogate markers suggests a favorable signal for sotatercept in this population. It also highlights its potential role in delaying the need for advanced therapies, or as a bridge to intervention through better PAH optimization. While early results are promising, further investigation through prospective registries or ACHD-specific sub-studies of ongoing sotatercept trials is needed.

Future avenues of exploration include whether sotatercept may allow for therapeutic possibilities previously unavailable to congenital patients. For instance, some shunts previously deemed inoperable due to prohibitive PVR could theoretically become amenable to full or partial closure following hemodynamic improvement on sotatercept, ie as part of a “treat-to-close” strategy [5], [6]. This holds special relevance in residual ASD or VSD scenarios where closing the defect may prevent long-term cyanosis, paradoxical embolism, or arrhythmia [5]. Certain complex congenital conditions such as single ventricle physiology, particularly those with a Fontan circulation, were not assessed. However, it is well-described that elevated PVR is a key driver of Fontan failure [7], and future studies would be needed to explore whether sotatercept could support failing Fontan physiology.

## 8 Conclusions

Sotatercept may offer a valuable addition to the treatment armamentarium for ACHD-PAH. In select patients, it may delay right-sided heart failure progression, improve symptoms, reverse adverse subpulmonary ventricular remodeling, and potentially defer advanced therapies such as lung transplantation. Patients with pulmonary veno-occlusive disease or with late-stage disease may derive limited benefit. Future studies are needed to assess its role in ACHD-PAH patients, especially in complex congenital disease such as single-ventricle physiology which has not yet been studied.

## CRediT authorship contribution statement

**Hans Gao:** Conceptualization, Formal analysis, Writing – original draft, Writing – review & editing. **Richard Channick:** Writing – review & editing. **Rajan Saggar:** Writing – review & editing. **Michael Troy:** Writing – review & editing. **Jeannette Lin:** Writing – review & editing. **Gentian Lluri:** Writing – review & editing. **Leigh Reardon:** Writing – review & editing. **Jamil Aboulhosn:** Conceptualization, Supervision, Writing – review & editing.

## Funding

“This research received no specific grant from any funding agency in the public, commercial, or not-for-profit sectors.”

## Declaration of competing interest

The authors declare the following financial interests/personal relationships which may be considered as potential competing interests: Rajan Saggar reports a relationship with United Therapeutics Corporation that includes: consulting or advisory, funding grants, and speaking and lecture fees. Rajan Saggar reports a relationship with Merck & Co Inc that includes: funding grants and speaking and lecture fees. Rajan Saggar reports a relationship with Liquidia Corporation Inc that includes: funding grants and speaking and lecture fees. Rajan Saggar reports a relationship with Gossamer Bio Inc that includes: consulting or advisory. Rajan Saggar reports a relationship with Janssen Pharmaceuticals Inc that includes: consulting or advisory. Rajan Saggar reports a relationship with Pulmovant Inc that includes: consulting or advisory. Rajan Saggar reports a relationship with Insmed Incorporated that includes: consulting or advisory. Rajan Saggar reports a relationship with Aria Pharmaceuticals that includes: consulting or advisory. Jamil Aboulhosn reports a relationship with Edwards Lifesciences Corporation that includes: consulting or advisory, funding grants, speaking and lecture fees, and travel reimbursement. Jamil Aboulhosn reports a relationship with Medtronic that includes: consulting or advisory, speaking and lecture fees, and travel reimbursement. Jamil Aboulhosn reports a relationship with Abbott that includes: consulting or advisory, speaking and lecture fees, and travel reimbursement. Richard Channick reports a relationship with Merck & Co Inc that includes: board membership, consulting or advisory, and travel reimbursement. Richard Channick reports a relationship with Bayer Corporation that includes: board membership, consulting or advisory, and speaking and lecture fees. Richard Channick reports a relationship with Gossamer Bio Inc that includes: board membership, consulting or advisory, and travel reimbursement. Richard Channick reports a relationship with United Therapeutics Corporation that includes: consulting or advisory. Richard Channick reports a relationship with Third Pole that includes: consulting or advisory. Leigh Reardon reports a relationship with Janssen Pharmaceuticals Inc that includes: speaking and lecture fees. “Given his role as editor in IJC CHD, Jamil Aboulhosn had no involvement in the peer review of this article and had no access to information regarding its peer review. Full responsibility for the editorial process for this article was delegated to another journal editor.” If there are other authors, they declare that they have no known competing financial interests or personal relationships that could have appeared to influence the work reported in this paper.
